# SALL1 as a marker for distinguishing microglia from blood-derived myeloid cells in stroke brain scRNA-seq data

**DOI:** 10.1186/s13024-026-01003-6

**Published:** 2026-10-15

**Authors:** Chu-Yan Zhang, Yi-Ming Lu, Heng Zhang, Yi-Sheng Li, Luo-Yi-Fan He, Yi-Yao Gao, Mu-Zhou Xue, Zi-Yu Wang, Zi-Bo Tang, Li-Li Feng, Jiali Wang, Yi-Xuan Zhang

**Affiliations:** 1 Medical Basic Research Innovation Center for Cardiovascular and Cerebrovascular Diseases, Ministry of Education, China, School of Pharmacy, Nanjing Medical University, Nanjing, China; 2 Department of Hematology and Oncology, Children’s Hospital of Nanjing Medical University, Nanjing, China; 3 Children’s Hospital of Nanjing Medical University, Nanjing, China; 4 International Joint Laboratory for Drug Target of Critical Illnesses, School of Pharmacy, Nanjing Medical University, Nanjing, China; 5 Northern Jiangsu Institute of Clinical Medicine, The Affiliated Huaian No.1 People’s Hospital of Nanjing Medical University, Huaian, China

**Keywords:** Stroke, Marker gene, Microglia, Myeloid cells, PBMCs, ScRNA-seq, SALL1

## Abstract

Distinguishing resident microglia from infiltrating myeloid cells in stroke scRNA-seq data remains challenging due to shared myeloid markers; SALL1 shows greater specificity for microglia compared with commonly used markers (e.g. TMEM119, CX3CR1, P2RY12), and combining SALL1 with these commonly used markers improves the ability to distinguish microglia from infiltrating myeloid populations in stroke‑associated scRNA‑seq datasets.

**Supplementary Information:**

The online version contains supplementary material available at https://doi.org/10.1186/s13024-026-01003-6.

Microglia, the major resident macrophages of the central nervous system (CNS), regulate neurodevelopment, synaptic remodeling, and neuroinflammatory regulation. Stroke-induced blood–brain barrier disruption enables extensive infiltration of peripheral myeloid cells (e.g., monocytes and macrophages) into the brain parenchyma [1]. Similar infiltration arises in CNS infections, traumatic brain injury, and even severe neurodegenerative diseases. These infiltrating cells interact with microglia to shape disease progression and recovery [2]. Although microglia and infiltrating myeloid cells can be distinguished by flow cytometry, their separation in single-cell RNA sequencing (scRNA-seq) or spatial transcriptomics is challenging due to shared transcriptional programs rooted in their common myeloid lineage.

Canonical markers widely used for scRNA-seq microglia annotation, including TMEM119, P2RY12, and CX3CR1, lack absolute specificity: TMEM119 can be downregulated in pathology [3], CX3CR1 is highly expressed in monocytes [4], and P2RY12 is detectable in platelets and T cells [5]. In physiological conditions with an intact BBB, this lack of absolute specificity has minimal impact since peripheral myeloid cells are largely absent from the CNS. In stroke, however, shared marker expression between resident and infiltrating myeloid cells produces annotation ambiguity, often leading to misclassification of infiltrating macrophages as microglia. To address this, we systematically evaluated microglial markers from published stroke datasets to identify genes that accurately discriminate resident microglia from infiltrating myeloid cells.

We analyzed 19 stroke scRNA-seq and spatial transcriptomics datasets from 17 studies (STable 1) and cataloged the top 10 markers most commonly used for microglia annotation (Fig. 1a). *Tmem119*, *Cx3cr1*, and *P2ry12* emerged as the most frequently employed, with combinatorial strategies often pairing *Tmem119* and *P2ry12*, supplemented by *Cx3cr1* or *Hexb* (Fig. 1b). Based on usage frequency, prevalence in multi-marker annotation schemes, and reported association with microglia, we selected *Tmem119*, *P2ry12*, *Cx3cr1*, and *Hexb* for specificity assessment. At the mRNA level, the human protein atlas (HPA), Monaco [6], and Schmiedel [7] datasets consistently demonstrated expression of all four markers in monocytes, dendritic cells, and granulocytes (Fig. 1c), confirming their limited specificity for distinguishing microglia from infiltrating myeloid cells in stroke. Using immunohistochemistry data from the HPA, all four markers were detectable in human peripheral immune organs—TMEM119, P2RY12, and CX3CR1 showed measurable expression in peripheral myeloid cells, while HEXB was even more abundantly expressed in peripheral tissues than in microglia (Fig. 1d, SFig. 1a).

**Fig. 1:**
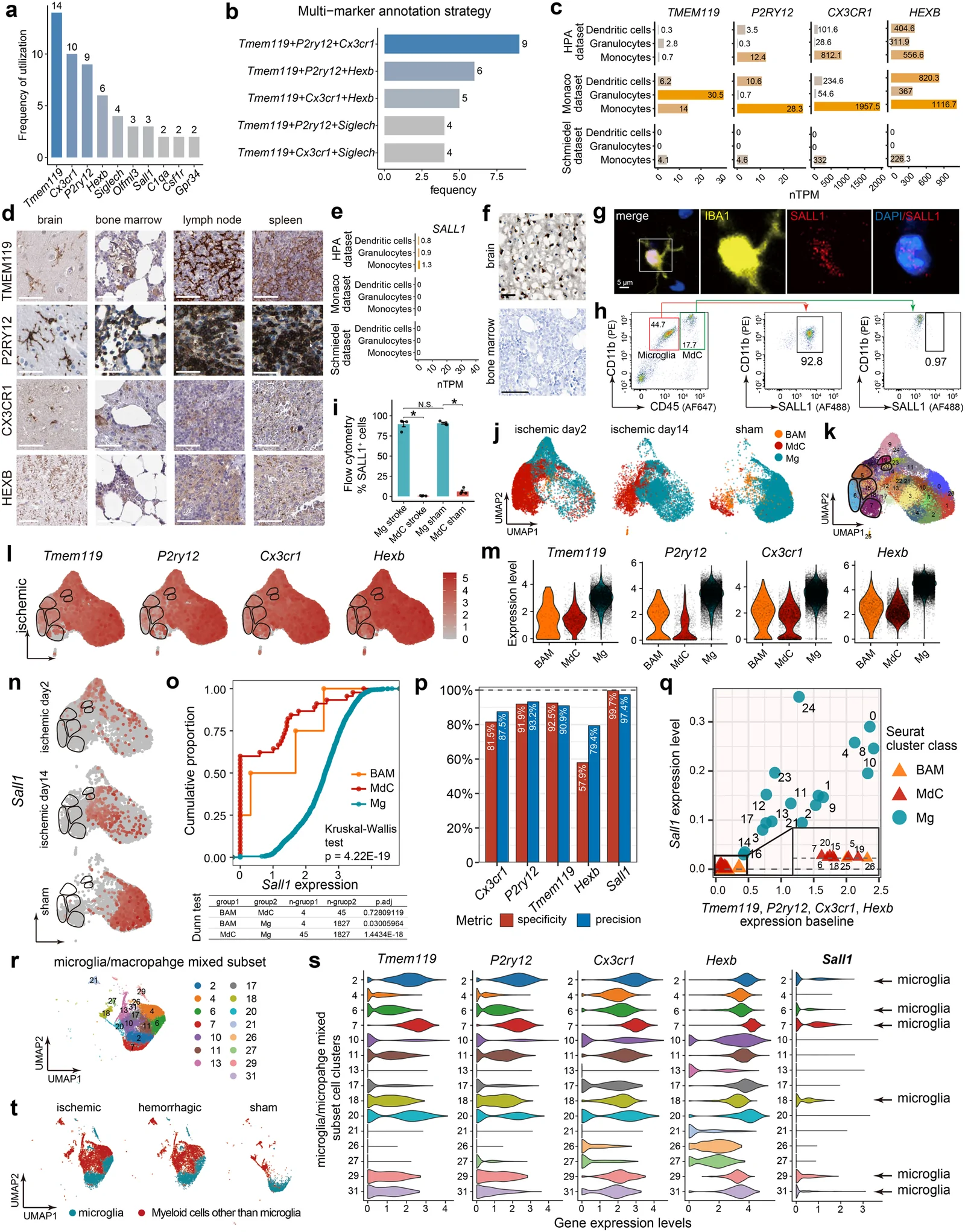
SALL1 as a marker for distinguishing microglia from blood-derived myeloid cells in stroke brain scRNA-seq data. **A.** Frequency of utilization of the top 10 microglial markers across 19 stroke-related datasets. To unify the gene symbols of different organisms, human marker genes were converted into homologous mouse genes and merged for statistical analysis. **B.** Frequency of microglia annotation strategies employing multi-marker combinations across 19 published stroke-related datasets. **C.** Top four microglia marker gene expression among peripheral myeloid cells from HPA database, the Monaco dataset and the Schmiedel dataset. nTPM: normalized transcripts per million. **D.** Top four microglia marker protein expression among brain and immune organs used the immunohistochemistry data from the HPA database. Scale bar: 25 μm. **E.** Gene expression levels of *SALL1*, integrating data from the HPA database, the Monaco dataset, and the Schmiedel dataset. **F.** Immunohistochemistry illustrating SALL1 protein expression. Scale bar: 50 μm. **G.** Immunofluorescent staining demonstrating the co-localization of SALL1 and IBA1 in ramified microglia within the mouse brain tissue. Scale bar: 5 μm. **H.** Flow cytometry gating strategy and SALL1 expression in microglia versus monocyte-derived cells (MdC) from hemorrhagic stroke brain. **I.** Flow cytometric quantification of SALL1^+^ cells across cell populations. Mg: microglia. **J.** Uniform manifold approximation and projection (UMAP) illustrating the distribution of myeloid cells under ischemic stroke and sham conditions. **K.** UMAP visualization delineating distinct subclusters of all cells. MdC are circled out. **L.** Log normalized expression levels of the top four commonly used microglia markers within this dataset, highlighting their observed expression within MdCs of clusters 5, 6, 7,15, 19, and 20. **M.** Violin plot depicting the log normalized expression levels of the top four microglia marker across all cell types within this dataset. **N.** Gene expression specificity of *Sall1* within microglia of this dataset, Log normalized. **O.** Empirical cumulative distribution function (ECDF) of *Sall1* Log normalized expression. *Sall1* levels were significantly higher in microglia (Kruskal–Wallis test and Dunn’s post hoc test with FDR). **P.** Bar plot comparing specificity and precision of various marker genes for microglia identification. **Q.** Dot plot showing the cluster average Log normalized expression levels of *Sall1* and other marker genes across distinct cell clusters. **R.** Clustering results of mixed myeloid cell populations within main study dataset. **S.** Gene expression of *Sall1* and the top four microglia markers across these myeloid cell clusters in the main study dataset, Log normalized. **T.** Re-annotation of previously mixed myeloid cell subpopulations, employing the *Sall1*^high^*Tmem119*^+^*P2ry12*^+^ combination strategy for enhanced microglia identification in the main study dataset

SALL1, a conserved transcription factor essential for microglial identity, shows negligible protein expression outside microglia despite low mRNA levels in neurons and oligodendrocytes [8]. Analysis of the HPA, Monaco, and Schmiedel datasets confirmed that *SALL1* is predominantly expressed in microglia, with minimal expression in peripheral myeloid lineages—including monocytes, macrophages, granulocytes, or dendritic cells (Fig. 1e). Immunohistochemistry in mice revealed strong SALL1 nuclear staining in brain but no signal in bone marrow (Fig. 1f), while immunofluorescence showed clear co-localization of SALL1 with IBA1 in microglial nuclei (Fig. 1g, SFig. 1b), We also performed flow cytometry on brain cells isolated from sham and stroke mice at 24 h post-injury. SALL1 protein was highly enriched in the CD11b^+^CD45^int^ microglial population, with over 90% of this population positive for SALL1. In contrast, fewer than 10% of CD11b^+^CD45^hi^ infiltrating peripheral myeloid-derived cell (MdC) expressed detectable SALL1 protein (Fig. 1h, i, SFig. 2a-d).

To validate SALL1’s specificity in a stroke context, we analyzed a dataset [9] comprising scRNA-seq of CD45^int^ and CD45^high^ immune cells, which were separately isolated by FACS from brain samples 2 and 14 days after ischemic stroke. The scRNA-seq data were processed using the standard Seurat pipeline, and batch effects were corrected using Harmony. Based on the cell type annotations provided in the original study [9], microglia, border-associated macrophage (BAM) and MdC were extracted from the original annotations to serve as test benchmark data (Fig. 1j, k). Canonical microglial markers—including *Tmem119*, *P2ry12*, *Cx3cr1*, and *Hexb*—were expressed across MdC clusters 5, 6, 7, 15, 19, and 20 (Fig. 1l), indicating that these genes are not exclusive to microglia (Fig. 1m). In contrast, *Sall1* was predominantly expressed in microglia clusters and largely absent from MdC clusters (Fig. 1n). Although *Sall1* expression displays microglial subcluster‑specific fluctuations over the post‑stroke time course, its overall expression is maintained in microglial populations. (SFig. 3a, b), supporting its utility for distinguishing between these populations.

To quantitatively evaluate Sall1 expression across myeloid subsets, we first performed an empirical cumulative distribution function (ECDF) analysis for Sall1-detectable cells. Among these cells, *Sall1* expression was significantly higher in microglia than in both BAM and MdC (Fig. 1o). Building on this, we next assessed *Sall1* as a binary classifier at the single-cell level by applying a fixed biological detection threshold. Confusion matrix analysis revealed that *Sall1* achieves higher specificity and precision (both > 97%) compared with other canonical microglial marker genes (Fig. 1p). We found that integrating *Sall1* with established microglial markers further improved annotation performance, yielding enhanced specificity and precision relative to conventional multi‑marker annotation strategies (SFig. 3c, d). Finally, to generalize these findings to the cell cluster level, we computed a baseline marker score—defined as the mean expression of *Tmem119*, *P2ry12*, *Cx3cr1*, and *Hexb*—for each cell cluster. While score was comparable across microglia, BAM, and MdC clusters, *Sall1* expression was selectively enriched in microglia and virtually absent in BAM and MdC, with non-microglial populations largely confined to the *Sall1*-negative zone (Fig. 1q). These results collectively establish *Sall1* as an incremental signature that discriminates microglia from other myeloid cells above the shared baseline.

To thoroughly evaluate the utility of Sall1 as a marker for distinguishing microglia from peripheral myeloid-derived cells, we analyzed a stroke scRNA-seq datasets [10] encompassing both ischemic and hemorrhagic stroke subtypes as our main study dataset (SFig. 4a-c). We further applied a combinatorial strategy—*Sall1*^high^, *Tmem119*^+^, *P2ry12*^+^—to re-analyze the dataset in which microglia and macrophages had originally co-clustered into a “microglia/macrophage” population (SFig. 4d). By increasing clustering resolution, we resolved the “microglia/macrophage” population into 15 subclusters (Fig. 1r), only six subclusters exhibited high *Sall1* expression and were confidently annotated as microglia (Fig. 1s). The refined microglia annotations were then projected onto the UMAP visualization (Fig. 1t), which revealed a clear and consistent distribution pattern: microglia from the sham group. To confirm the performance of *Sall1* across diverse scRNA-seq platforms and sequencing instruments, we generated and analyzed an independent stroke dataset using technologies distinct from those employed for the benchmark and main study datasets (SFig. 5a). We found that *Sall1* retained cluster-specific expression relative to other marker genes (SFig. 5b, c).

Nevertheless, several limitations of the present study should be noted. Our conclusions are drawn from one benchmark dataset and two independent validation datasets. The influences of multiple variables remain incompletely evaluated, including the diversity of stroke brain samples (e.g., distinct animal stroke‑induction methods, different experimental species, and varied brain regions from human stroke specimens), pre‑sequencing sample quality control (such as RNA degradation, freeze‑thaw artifacts, and sample contamination), and scRNA-seq platform and sequencing technologies beyond those shown in SFig. 5a. Despite these limitations, our findings confirm that combining *Sall1* with established markers improves the performance to distinguish microglia from peripheral myeloid‑derived cells in stroke‑associated scRNA‑seq datasets.

## Electronic supplementary material

Below is the link to the electronic supplementary material.

Supplementary Material 1

Supplementary Material 2

Supplementary Material 3

## Data Availability

The data supporting the findings of this study are included in the main article and Supplementary material. Additional codes are available from the corresponding author upon reasonable request.
