# Supplementary material for: SALL1 as a marker for distinguishing microglia from blood-derived myeloid cells in stroke brain scRNA-seq data
Source: Mol Neurodegener. 2026 Oct 15;21:67. doi: 10.1186/s13024-026-01003-6 (PMC13673770; doi:10.1186/s13024-026-01003-6)
Supplement: Supplementary file 1 [file 13024_2026_1003_MOESM1_ESM.docx]

**Methods and materials**

**Animal care**

Animal care and experimental procedures were approved by the Animal Welfare Ethics Committee of Nanjing Medical University (Ethics No. IACUC-2206009-2) in compliance with Chinese animal welfare laws. All animal care and experimental procedures adhered to the National Institutes of Health Guide for the Care and Use of Laboratory Animals.

**Human gene and protein expression levels**

Human gene expression levels across cell types and brain regions were obtained from the human protein atlas (HPA) database and are represented as normalized transcripts per million (nTPM). In HPA, raw transcript per million (TPM) values for each sample were first scaled to a total of 1 million, yielding pTPM (protein-coding TPM), to account for the prior exclusion of non-coding transcripts. Subsequently, within each data source, pTPM values across all samples were normalized using the trimmed mean of M-values (TMM) method to enable robust between-sample comparisons. The final normalized expression values, denoted nTPM, were computed for each gene in every sample. Human protein expression in various organs was assessed by immunohistochemistry using data from the HPA tissue atlas.

**Human gene expression levels in external immune cell RNA-seq data**

Human gene expression levels in external immune cell RNA-seq datasets were harmonized with the HPA for cross-dataset comparability. In the Schmiedel dataset, gene-level nTPM values for immune cell types were mapped to the Ensembl gene annotation version used by HPA. In the Monaco dataset, raw sequencing reads were processed through the same RNA-seq analysis pipeline as HPA-generated data and normalized using the TMM method to enable between-sample comparisons. The resulting expression values were reported as nTPM.

**Mouse hemorrhagic stroke model induction**

C57BL/6J mice (8–10 weeks old, 25–30 g) were obtained from Charles River (Wilmington, Massachusetts, USA). Mice were anesthetized with isoflurane (RWD, Shenzhen, China) and positioned in a stereotaxic frame. Autologous blood (20 μL) was collected from the tail artery and injected into the right striatum in two steps. Sham mice underwent identical surgical procedures without blood injection.

**Immunohistochemistry**

Immunohistochemistry was performed on 8 μm paraffin-embedded mouse brain and decalcified thighbone samples. Samples were deparaffinized, rehydrated, and subjected to antigen retrieval in citrate buffer (pH 6.0), followed by incubation with 3% hydrogen peroxide and blocking with 3% goat serum. Sections were then incubated overnight at 4 °C with primary antibody against Sall1 (Proteintech, #27761-1-AP). After washing with PBS, HRP-conjugated secondary antibody was applied for 50 min at room temperature. Signals were visualized using DAB substrate, and nuclei were counterstained with hematoxylin.

**Immunofluorescence**

Brain samples were embedded in paraffin, and sectioned at 8 μm thickness using a rotary microtome (Leica RM2235, Germany). Prior to immunostaining, sections were deparaffinized in xylene and rehydrated through a graded ethanol series to distilled water. Antigen retrieval was performed, followed by cooling to room temperature for 20 min. Sections were then permeabilized with 0.3% Triton X-100 in PBS for 15 min and blocked with 5% normal donkey serum in PBS for 1 h at room temperature. Primary antibodies—SALL1 (Proteintech, #27761-1-AP), and IBA1 (abcam, #ab5076)—were diluted in blocking buffer and applied overnight at 4 °C. After washes, sections were incubated with fluorescently labeled secondary antibodies for 2 h at room temperature in the dark. Following additional PBS washes, sections were mounted with ProLong™ Gold Antifade Mountant with DAPI. Fluorescent images were acquired using a slide scanning microscope (ZEISS Axioscan 7, Germany) and processed with ZEN v3.10 (ZEISS, Germany).

**Flow cytometry**

Mice were anesthetized and perfused with ice cold phosphate buffered saline (PBS). Brains were rapidly removed, and the tissue was minced with scissors into small pieces. The minced tissue was then incubated in digestion solution at 37 °C for 30 min with gentle inversion every 10 min. The digested suspension was filtered through a 70 μm cell strainer to remove debris and centrifuged at 1,200 × g for 5 min to pellet cells. The pellet was washed once with cold PBS and resuspended with 4 mL 30 % Percoll solution and centrifuged at 800 × g for 30 min with low brake. The cell pellet was collected and washed once with PBS.

The resulting single cell suspension was stained with the following antibodies at 4 °C in the dark for 30 min: CD45 AF647 (BioLegend, #103124), CD11b PE (eBioscience, #12 0112 81), and Fixable Viability Stain 510 (BD Horizon, #564406). Subsequently, cells were subjected to fixation and permeabilization using the Intracellular Fixation & Permeabilization Buffer Set (eBioscience #88 8824 00), followed by FcR blockade. Intracellular staining was then performed with SALL1 AF488 antibody (eBioscience, #53 9729 80) at 4 °C for 30 min. Finally, stained cells were acquired on a BD FACSymphony A5 flow cytometer (BD, USA) for analysis.

**Single cell RNA-seq data analysis of benchmark and main study dataset**

The FACS-sorted scRNA-seq dataset from ischemic stroke and sham mice was obtained from GSE225948. Additionally, the scRNA-seq data used in the SALL1 annotation strategy application—including ischemic stroke, hemorrhagic stroke, and sham mice—was sourced from GSE167593. scRNA-seq data quality control, normalization, and dimensionality reduction were performed using Seurat (v5.0.1). Within each dataset, batch effects across multiple samples were corrected using Harmony (v1.2.0). The two datasets were analyzed independently, and no cross-dataset integration or joint analysis was performed. All downstream marker specificity and precision analyses were conducted separately within each dataset.

**Mouse hemorrhagic stroke single-cell RNA sequencing**

Brain was collected from hemorrhagic stroke mice at days 1 post-induction and from sham mice. Tissues were dissociated into single-cell suspensions and processed on the MobiNova platform (OE Biotech, Shanghai, China). Reverse transcription, cDNA amplification, and library construction were performed according to the manufacturer's protocol. Libraries were sequenced on the DNBSEQ-T7 platform (MGI, Shenzhen, China). Raw FASTQ files were aligned to the GRCm39 reference genome. scRNA-seq data were processed using Seurat (v5.0.1). Harmony (v1.2.0) is utilized for eliminating batch effects. Doublets were removed with DoubletFinder. Barcodes were retained if they contained 50–7,500 genes, <15% mitochondrial reads, and <5% erythrocyte gene expression.

**Homologous gene conversion between humans and mice**

Human gene annotation information was obtained from org.Hs.eg.db (v3.18.0), mouse gene annotation information was obtained from org.Mm.eg.db (v3.18.0), and homologous gene conversion between human and mouse was performed using homologene (v1.4.68).

**Gene expression distribution and pairwise statistical comparisons of Sall1 across microglial subclusters and experimental groups**

Single-cell expression levels of *Sall1* were profiled across microglial subclusters and experimental groups (Sham, D02, and D14). Mean *Sall1* expression values were calculated for each subcluster under each experimental condition and visualized as a row-scaled heatmap to illustrate relative expression changes across time points within individual subclusters. The single-cell distribution of *Sall1* expression was further visualized using boxplots. Pairwise statistical comparisons of expression levels among experimental groups were performed within each microglial subcluster using the Wilcoxon rank-sum test.

**Marker specificity and precision evaluation**

For each candidate marker gene, we extracted its log normalized expression profile across all retained cells and applied a fixed detection threshold of expression more than zero to generate a binary prediction:

$${\overset{^}{y}}_{i,g}=1(x_{i,g}>0)$$

where $x_{i, g}$ is the normalized expression of gene *g* in cell *i*, and ${\overset{^}{y}}_{i,g}=1$ indicates a predicted positive call. Ground-truth labels were defined by the annotated cell population, with microglia $celltype=Mg$ as the positive class and border-associated macrophages (BAM) and monocyte-derived cells (MdC) as the negative class:

$$y_{i}=\{\begin{matrix} 1 & {if celltype}_{i}=Mg \\ 0 & otherwise \end{matrix}$$

From these, we constructed a confusion matrix at the single-cell level by counting true positives (TP), false positives (FP), false negatives (FN), and true negatives (TN):

$$TP=\sum_{i=1}^{n} 1({\overset{^}{y}}_{i,g}=1\wedge y_{i}=1)$$

$$FP=\sum_{i=1}^{n} 1({\overset{^}{y}}_{i,g}=1\wedge y_{i}=0)$$

$$FN=\sum_{i=1}^{n} 1({\overset{^}{y}}_{i,g}=0\wedge y_{i}=1)$$

$$TN=\sum_{i=1}^{n} 1({\overset{^}{y}}_{i,g}=0\wedge y_{i}=0)$$

We quantified marker performance using specificity (true negative rate) and precision (positive predictive value, PPV):

$$Specificity=\frac{TN}{TN+FP}$$

$$Precision=\frac{TP}{TP+FP}$$

Based on the above single‑marker evaluation framework, we further assessed the annotation performance of four multi‑marker combinatorial strategies for microglia identification. Following identical binary expression thresholds, a cell was defined as positive for a given marker panel only when each marker gene in the panel independently met the expression cutoff (> 0). The same confusion‑matrix calculation and performance‑metric formulas described above were uniformly applied to evaluate the specificity and precision of each multi‑marker panel.

**Cluster-level marker quantification**

To mitigate single-cell dropout and obtain robust population-level estimates, we computed the mean normalized expression (log-normalized) for each gene of interest within every Seurat cluster *c*:

$${\overset{¯}{X}}_{g,c}=\frac{1}{n_{c}}\sum_{i=1}^{n_{c}} x_{g,i}$$

where $x_{g,i}$ denotes the expression of gene *g* in cell *i*, and $n_{c}$ is the total number of cells in cluster *c*. Cluster identity was assigned by majority vote of the annotated cell population (Mg, BAM, or MdC).

***Tmem119, P2ry12, Cx3cr1, Hexb* expression baseline**

We defined a myeloid background (MB) score to capture the *Tmem119, P2ry12, Cx3cr1, Hexb* expression baseline that is present across microglia, BAM, and MdC:

$${MB}_{c}=\frac{1}{4}({\overset{¯}{X}}_{Tmem119,c}+{\overset{¯}{X}}_{P2ry12,c}+{\overset{¯}{X}}_{Cx3cr1,c}+{\overset{¯}{X}}_{Hexb,c})$$

Cluster-level relationships between myeloid background and *Sall1* expression were visualized as scatter plots, with point color indicating the cell type and point size scaled to *Sall1* expression level.

**Data visualization and statistics**

Data was visualized by ggplot2 (v3.5.0) and ComplexHeatmap (v2.15.4). The statistical analyses were conducted using R (v 4.3.2) and rstatix (v 0.7.2).
