# Supplementary material for: SALL1 as a marker for distinguishing microglia from blood-derived myeloid cells in stroke brain scRNA-seq data
Source: Mol Neurodegener. 2026 Oct 15;21:67. doi: 10.1186/s13024-026-01003-6 (PMC13673770; doi:10.1186/s13024-026-01003-6)
Supplement: Supplementary file 2 [file 13024_2026_1003_MOESM2_ESM.docx]

**Supplementary figure 1 Microglia marker gene expression.**

**(a)** Top four microglia marker genes expression among myeloid cells used the average normalized transcripts per million (nTPM) data from the HPA database.

**(b)** Immunofluorescent staining demonstrating the co-localization of SALL1 and IBA1 in amoeboid microglia within the mouse brain tissue. Scale bar: 5μm.

**Supplementary figure 2. Flow cytometry analysis of SALL1 protein expression in brain myeloid cells from control and hemorrhagic stroke model mice.**

**(a, b)** Gating strategy for identifying CD11b^+^CD45^int^ microglia and CD11b^+^CD45hi infiltrating MdC in control (a) and hemorrhagic stroke model (b) mouse brain samples, followed by quantification of SALL1-positive populations within each gate.

**(c, d)** Contour plots highlighting SALL1-positive cells (blue dots) among CD11b^+^ myeloid subsets in control (c) and hemorrhagic stroke model (d) groups.

**Supplementary Figure 3. *Sall1* expression profiles and performance of multi‑marker strategies for microglia annotation.**

**(a)** Box plots showing the normalized *Sall1* expression levels across microglial subclusters in the sham, day 2, and day 14 groups after ICH. Wilcoxon rank-sum test.

**(b)** Heatmap illustrating the clustering patterns of *Sall1* expression across microglial subclusters among Sham, day 2, and day 14 ICH groups.

**(c)** Bar plot comparing annotation performance between the *Sall1* + *Tmem119* + *P2ry12* panel and other commonly‑used multi‑marker strategies for microglia annotation.

**(d)** Bar plot illustrating classification performance of multi‑marker panels combining *Sall1* with other well‑established microglia marker genes.

**Supplementary figure 4 SALL1-based microglia identification in stroke scRNA-seq data.**

**(a)** Workflow illustrating the performance evaluation of the *Sall1*^high^ *Tmem119*^+^ *P2ry12*^+^ combination strategy for distinguishing microglia from other myeloid cells within stroke-related sc/snRNA-seq datasets.

**(b)** UMAP visualization of cell clustering results of scRNA-seq dataset GSE167593 from Yang and Tang et al study.

**(c)** Dot plot illustrating the canonical marker expression in each cell type.

**(d)** UMAP visualization of cell annotation results, demonstrating a close resemblance to the overall dataset structure as presented in the original publication by Yang and Tang et al.

**Supplementary figure 5** **Cross-platform validation confirming the stable discriminatory performance of Sall1.**

**(a)** Table summarizing the scRNA-seq platforms and sequencing instruments for the benchmark, main study and independent validation datasets.

**(b)** UMAP visualization of cell clusters derived from our independent stroke scRNA-seq dataset acquired on a separate platform.

**(c)** Violin plots displaying expression levels of *Sall1* and other myeloid marker genes across individual cell clusters.
